# Brother of the regulator of the imprinted site (BORIS) variant subfamily 6 is a novel target of lung cancer stem-like cell immunotherapy

**DOI:** 10.1371/journal.pone.0171460

**Published:** 2017-03-01

**Authors:** Ryota Horibe, Yoshihiko Hirohashi, Takuya Asano, Tasuku Mariya, Takeshi Suzuki, Akari Takaya, Hiroshi Saijo, Yosuke Shionoya, Terufumi Kubo, Munehide Nakatsugawa, Takayuki Kanaseki, Tomohide Tsukahara, Kazue Watanabe, Eri Atsuyama, Shingo Toji, Hiroshi Hirano, Tadashi Hasegawa, Hiroki Takahashi, Noriyuki Sato, Toshihiko Torigoe

**Affiliations:** 1 Department of Pathology, Sapporo Medical University School of Medicine, Japan; 2 Department of Respiratory Medicine and Allergology, Sapporo Medical University School of Medicine, Japan; 3 Department of Obstetrics and Gynecology, Sapporo Medical University School of Medicine, Japan; 4 Department of Biology, Sapporo Medical University School of Medicine, Japan; 5 MEDICAL and BIOLOGICAL LABORATORIES CO., LTD., Japan; 6 Department of Surgical Pathology, Sapporo Medical University School of Medicine, Japan; Okayama University, JAPAN

## Abstract

Lung cancer is one of the most common malignancies with a high rate of mortality. Lung cancer stem-like cells (CSCs)/ cancer-initiating cells (CICs) play major role in resistance to treatments, recurrence and distant metastasis and eradication of CSCs/CICs is crucial to improve recent therapy. Cytotoxic T lymphocytes (CTLs) are major effectors of cancer immunotherapy, and CTLs recognize antigenic peptides derived from antigens that are presented by major histocompatibility complex (MHC) class I molecules. In this study, we analyzed the potency of a cancer-testis (CT) antigen, brother of the regulator of the imprinted site variant subfamily 6 (BORIS sf6), in lung CSC/CIC immunotherapy. BORIS sf6 mRNA was expressed in lung carcinoma cells (9/19), especially in sphere-cultured lung cancer stem-like cells, and in primary lung carcinoma tissues (4/9) by RT-PCR. Immunohistochemical staining using BORIS sf6-specific antibody revealed that high expression of BORIS sf6 is related to poorer prognosis. CTLs could be induced by using a human leukocyte antigen, (HLA)-A2 restricted antigenic peptide (BORIS C34_24(9)), from all of 3 HLA-A2-positive individuals, and CTL clone cells specific for BORIS C34_24(9) peptide could recognize BORIS sf6-positive, HLA-A2-positive lung carcinoma cells. These results indicate that BORIS sf6 is a novel target of lung cancer immunotherapy that might be useful for targeting treatment-resistant lung cancer stem-like cells.

## Introduction

Lung cancer is one of the most common malignant diseases worldwide and is the cause of leading cancer-associated death in both sexes in the U.S. and in other industrialized countries [1]. The five-year survival rate of lung cancer patients is only 70%, even if the cancer is diagnosed in an early stage [2], and the five-year survival rate of patients with advanced lung cancer is less than 15% [3]. Despite recent progress in chemotherapy, radiotherapy and molecular targeted therapy, the five-year survival rate has not been improved in the past several decades. The reasons for the poor prognosis are that lung cancer is frequently treatment-resistant and that the incidences of recurrence and metastasis are high.

Cancer stem-like cells (CSCs)/cancer-initiating cells (CICs) are defined as a small subpopulation of tumor cells that are endowed with high levels of tumor-initiating ability, self-renewal ability and differentiation ability. These cells are highly resistant to current cancer treatment, though CSCs/CICs are considered to be major causes of cancer recurrence, distant metastasis and treatment resistance [4, 5]. The presence of CSCs/CICs was first demonstrated in acute myeloid leukemia [6]. CSCs/CICs have since been found in several solid malignancies. In lung cancer, CSCs/CICs have been isolated by the side population (SP) assay [7, 8], Aldefluor assay [9] and use of cell surface markers including CD44 [10], CD133 [11] and CD166 [12], and it has been shown that lung CSCs/CICs are resistant to chemotherapy, radiotherapy and molecular targeting therapy [7, 13–16]. Therefore, a novel approach for eradication of lung CSCs/CICs is needed to improve the outcome of lung cancer.

Cancer immunotherapy is the fourth cancer treatment following surgery, chemotherapy and radiotherapy. A recent clinical study of revealed that anti-programmed death-1 (PD-1) antibody therapy showed benefits on patients prognosis than docetaxel and cancer immunotherapy is expected in lung cancers [17]. The effectors of cancer immunotherapy are cytotoxic T lymphocytes (CTLs) and CTLs recognize antigenic peptides presented by major histocompatible complex (MHC) class I. Thus, identification of antigenic peptides is essential to establish cancer immunotherapy.

In this study, we analyzed the expression of a cancer-testis antigen, brother of the regulator of the imprinted site variant subfamily 6 (BORIS sf6), in lung CSCs/CICs and non-CSCs/CICs, and we examined the potency of BORIS sf6 as a molecular target in lung CSC/CIC-targeting immunotherapy.

## Materials and methods

### Ethics statement

All studies were approved by the Institutional Review Board (IRB) of Sapporo Medical University Hospital (#25–214). Written informed consent was obtained from all patients and healthy blood donors according to the guidelines of the Declaration of Helsinki.

### Cell lines and cell culture

Human lung small cell carcinoma cell lines SBC1, SBC3, SBC5 and Lc817, lung squamous cell carcinoma cell line LK-2, and lung adenocarcinoma cell line PC3 were purchased from the Japanese Cancer Research Resources Bank (JCRB, Osaka, Japan). Human lung large cell carcinoma cell lines 86–2 and Lu99A and lung squamous cell carcinoma cell lines EBC1 and Sq-1 were obtained from the Cell Resource Center for Biomedical Research, Tohoku University (Sendai, Japan). Human lung adenocarcinoma cell lines LC81, LC142 and LC153 were kind gifts from Dr. K. Kuromuma (Sapporo Medical University School of Medicine, Sapporo, Japan) [18, 19]. The human lung adenocarcinoma cell line LHK2 was established in our laboratory [20]. The human lung adenocarcinoma cell line A549, lymphoblastoid cell line T2, transporter associated with antigen processing (TAP)-deficient, and the human erythroleukemia cell line K562 were purchased from American Type Culture Collection (ATCC, Manassas, VA, USA). Human primary lung adenocarcinoma cells (Primary #3, Primary #4, Primary #5 and Primary #7) were isolated and cultured from pleural effusion of lung cancer patients with written informed consent from the patients. SBC1, Lu99A, LK2, PC3, LC81, LC142 and LC153 cells were cultured in RPMI-1640 (R8758, Sigma-Aldrich, St Louis, MO, USA). SBC3, SBC5, 86–2, EBC1, Sq-1, A549, LHK2, Primary3, Primary4, Primary5 and Primary7 cells were cultured in Dulbecco's modified Eagle's medium (DMEM; 11965–092, Thermo Fisher Scientific, Yokohama, Japan). To all media, 10% fetal bovine serum (FBS)(FB-1345/500, biosera, Kansas City, MO, USA) was added. Cells were incubated in a humidified 5% CO_2_ incubator at 37°C.

### Sphere culture

A sphere formation assay was performed using a stem cell medium (11330–032, DMEM/F12 medium, Life Technologies) supplemented with 20 ng/mL human EGF (236-EG-200, R & D systems, Minneapolis, USA) and 20 ng/mL human basic fibroblast growth factor (233-FB-025, R & D systems, Minneapolis, USA) as described previously [21].

### RT-PCR analysis

Reverse transcription polymerase chain reaction (RT-PCR) was performed as described previously [22]. Total RNA (tRNA) was isolated from cultured cells by using an RNeasy Mini Kit (74104, Qiagen, Valencia, CA, USA), and tRNA was isolated from formalin-fixed, paraffin-embedded (FFPE) samples by using a NucleoSpin total RNA FFPE kit (740982–50, TaKaRa Bio., Kusatsu, JAPAN) according to the manufacturers’ protocols. cDNA was synthesized using Superscript III and oligo (dT) primer or random hexamer (Thermo Fisher Scientific) according to the manufacturer’s protocol. PCR amplification was performed with Taq DNA polymerase (Qiagen). PCR mixtures were initially incubated at 94°C for 2 min, followed by 40 cycles of denaturation at 94°C for 15 sec, annealing at 60°C for 30 sec and extension at 68°C for 30 sec. Primer pairs used for RT-PCR analysis using cDNA from cell lines were 5’-CTCAGGTAAGGGCTCTGGTG-3’ and 5’-TACTCCACACAGTGGGGTTG-3’ for *BORIS sf6* with an expected PCR product size of 220 base pairs (bps) and 5’-ACCACAGTCCATGCCATCAC-3’ and 5’-TCCACCACCCTGTTGCTGTA-3’ for *glyceraldehyde-3-phosphate dehydrogenase* (*GAPDH*) with an expected product size of 452 bps for cDNA from cell lines. Primer pairs used for RT-PCR analysis using cDNA from FFPE samples were 5’-CCATGTGCAAGTATGCCAGT-3’ and 5’- CTTCAGCACCAGAGCCCTTAC-3’ for *BORIS sf6* with an expected PCR product size of 168 bps and 5’-GAGTCAACGGATTTGGTCGT-3’ and 5’- TTGATTTTGGAGGGATCTCG-3’ for GAPDH with an expected product size of 238 bps. *GAPDH* was used as an internal control.

### Western blot

A Western blot was performed as described previously. [23] Adherent- and sphere-cultured SBC5 cells were lysed by an SDS sample buffer. Protein samples were applied to SDS-PAGE, and separated proteins were transferred onto a PVDF membrane (Immobion-P transfer membrane, Melck). After blocking with 5% skim milk in Tris-buffered saline containing 0.03% Tween20 (TBS-T) for overnight, the membrane was incubated with a primary antibodies in TBS-T containing 5% skim milk for 40 min at room temperature. Anti-SOX2 (AM2048a, ABGENT) was used at 500-times dilution and anti-β-Actin antibody (A2228, Sigma-Aldrich) was used at 2000-times dilution.

### Establishment of anti-BORIS sf6 polyclonal antibody and immunohistochemical staining

Anti-BORISsf6 polyclonal antibody was established by using synthetic peptide according to BORIS sf6 specific sequences (CSFKKLLFIGTIKVQR and KGSGAEGLIPTVLTLKASFKC) (Medical and Biological Laboratories Co., Ltd., Nagoya, JAPAN). The specificity of polyclonal antibody was confirmed by Western blot using BORIS sf5 overexpressed cells. Immunohistochemical staining was performed as described previously [24]. Anti-BORIS sf6 polyclonal antibody was used at 200 times dilution.

### CTL induction, Peptide-MHC tetramer assay and establishment of CTL clone

CTL induction using PHA blasts was performed as described previously [25]. CD8^+^ T cell reactivity was assessed by the interferon (IFN)-γ enzyme-linked immunospot (ELISpot) assay and tetramer assay. An IFN-γ ELISPOT assay was performed as described previously [26] using 1×10^4^ CTLs and 5×10^4^ T2 cells with each peptide (20 μg/mL). A Peptide-MHC tetramer assay was performed as described previously [27]. BORIS C34_24(9) peptide/HLA-A2 tetramers were synthesized by MBL (Nagoya, Japan). CTLs were stained by the tetramer at 4°C for 30 min at a 1:20 dilution and then stained for a further 20 min with fluorescein isothiocyanate (FITC)-conjugated mouse anti-CD8 monoclonal antibody (1:20 dilution). Stained CTLs were analyzed with a FACSaria II (BD Biosciences). To obtain CTL clones, tetramer-positive and single cell sorting was performed with FACSaria II.

### LDH release cytotoxicity assay

The antigen-specific lytic activity of CTL clones was evaluated using an LDH Cytotoxicity Detection Kit (MK401, Takara Bio, Otsu, Japan) according to the manufacturer’s protocol. Target cells (10000 cells/well) were incubated with various numbers of effector cells for 9 hours at 37°C in 96-well V-bottomed plates, and cytotoxicity was calculated using the culture supernatants. The percentage of specific lysis was calculated as [(experimental release − spontaneous release)/(maximum release − spontaneous release)] × 100.

### Sphere-formation inhibition assay

SBC5 cells or LC142 cells were cultured in sphere culture condition using 96 well ultra-low attachment plate (CLS3474, Sigma-Aldrich) (10^3^/well) for 6 days with 10^4^ of BORIS sf6 peptide specific CTL clone (3B9) or BORIS sf6 non-specific CTL clone (5H6). The numbers of spheres were counted and the data were analyzed statistically.

### Statistical analysis

Data for samples in the LDH release cytotoxicity assay were analyzed by Student’s t-test, with *P* < 0.05 conferring statistical significance. Chi-square test was used to determine the significance of associations between characteristic valuables. Survival curves were constructed according to the Kaplan–Meier method, and differences between groups were tested by the log-rank test. Statistical significance was determined by the log-rank test. P < 0.05 was considered significant. Statistical analysis was done with GraphPad Prism (version 4.0 for Windows; GrapgPad Software Inc).

## Results

### BORIS sf6 is highly expressed in lung cancer stem-like cells

**Fig 1:**
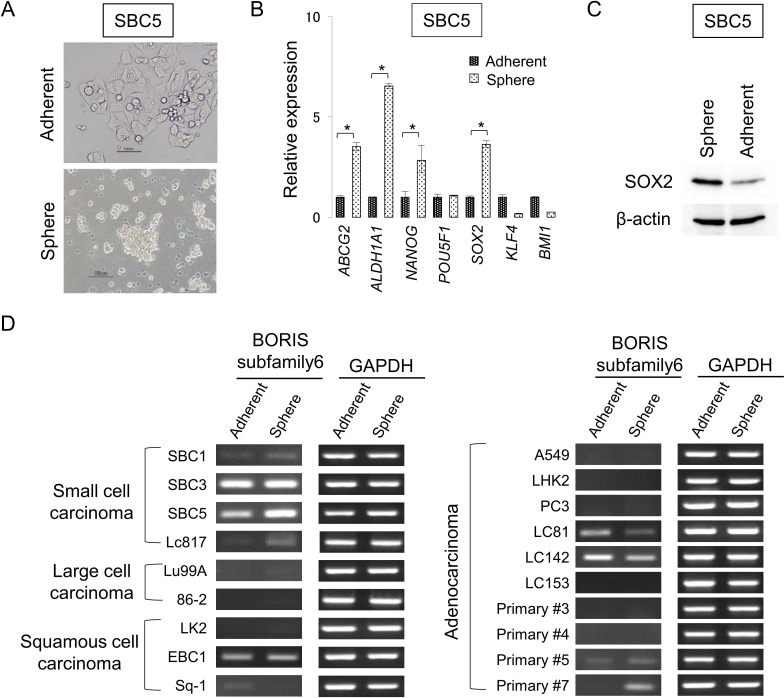
BORIS subfamily 6 expression in lung cancer cell lines. **(A) An image of sphere-cultured SBC5 cells.** Magnification, x 400. Bar scale is 100 μm. **(B) Expression levels of stem cell-related genes.** Expression levels of stem cell-related genes including ABCG2, ALDH1A1, NANOG, POU5F1, SOX2, KLF4 and BMI1 were determined by quantitative RT-PCR. Data are shown as means ± SD. All statistical analyses for data shown in this figure were performed using bilateral Student’s t test. *P-values <0.05. **(C) Expression of SOX2 protein in sphere-cultured cells.** SOX2 protein expression in sphere-cultured and adherent-cultured SBC5 cells were analyzed by an Western blot. β-Actin was used as a positive control. **(D) Expression of the cancer testis antigen BORIS sf6 in adherent-cultured and sphere-cultured lung carcinoma cells.** RT-PCR analysis of BORIS subfamily 6 mRNA expression in lung cancer cell lines and primary cancer cells from clinical specimens. Cancer cells were cultured in adherent culture and sphere culture.

Several methods for isolation of lung CSCs/CICs have been described, and a recent study revealed that lung CSCs/CICs were enriched in sphere-cultured cells [28]. We therefore cultured lung cancer cell lines derived from four major histological subtypes of lung carcinomas including small cell carcinomas (SBC1, SBC3, SBC5, Lc817), large cell carcinomas (Lu99A, 86–2), squamous cell carcinomas (LK2, EBC1, Sq-1) and adenocarcinomas (A549, LHK2, PC3, LC81, LC142, LC153, primary #3, primary #4, primary #5, primary #7) (Fig 1A). Quantitative RT-PCR (qRT-PCR) was performed to confirm that CSCs/CICs are enriched in sphere-cultured cells. Sphere-cultured cells derived from SBC5 cells showed high expression levels of stem cell-related markers including ABCG2, ALDH1A1, NANOG and SOX2 compared with the levels in adherent-cultured cells derived from SBC5 cells, indicating that sphere-cultured cells were enriched with CSCs/CICs (Fig 1B). Furthermore, SOX2 protein was preferentially expressed in sphere-cultured cells compared with adherent-cultured cells of SBC5 cells (Fig 1C).

In our previous study, we found that BORIS sf6 is preferentially expressed in cervical CSCs/CICs [27]. We therefore investigated the expression of BORIS sf6 in sphere-cultured cells. RT-PCR analysis showed that BORIS sf6 was expressed in adherent-cultured cells of 5 lung carcinomas (2 small cell carcinomas, 1 squamous cell carcinoma, 2 adenocarcinomas) and in sphere-cultured cells of lung carcinomas (4 small cell carcinomas, 1 squamous cell carcinoma, 4 adenocarcinomas) (Fig 1D). The results suggest that BORIS sf6 is expressed in some lung cancer cells and that the frequency of expression is higher in CSCs/CICs than in non-CSCs/CICs.

### BORIS sf6 is expressed in clinical specimens of lung cancer

**Fig 2:**
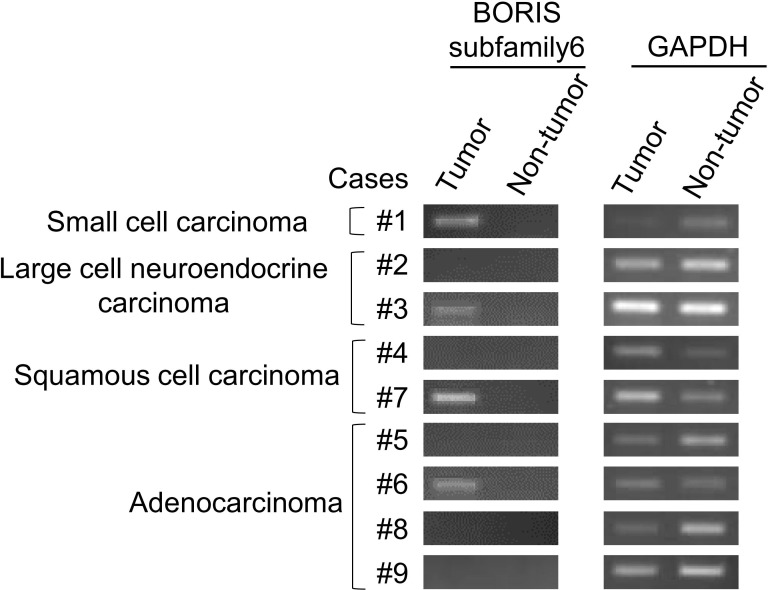
BORIS subfamily 6 expression in lung cancer clinical specimens. BORIS sf6 expression in several lung cancer tissue specimens was examined by RT-PCR. RNAs of tumor cells and non-tumor cells were extracted from formalin-fixed paraffin-embedded (FFPE) samples. GAPDH was used as an internal positive control.

Since BORIS sf6 was found to be expressed in sphere-cultured cells derived from primary lung adenocarcinomas (primary #5 and primary #7) (Fig 1C), we further analyzed the expression of BORIS sf6 in human lung carcinoma tissues using formalin-fixed paraffin-embedded (FFPE) tissues. RNA was isolated from lung carcinoma tissues and adjacent non-tumor lung tissues from 9 lung carcinoma cases. The RNA was analyzed by RT-PCR, and BORIS sf6 mRNA was detected in lung tissues in 4 of 9 cases but was not detected in adjacent non-tumor lung tissues (Fig 2).

**Fig 3:**
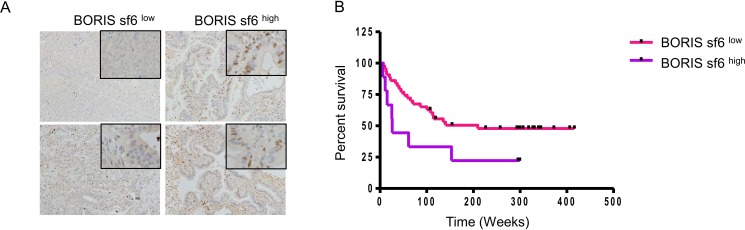
BORIS sf6 protein expression in lung cancer tissues. (A) Representative images of BORIS sf6^low^ and BORIS sf6^high^ case. Original magnification is 100 x. (B) Kaplan-Meier survival estimates were performed according to immunohistochemistry positivity of BORIS sf6. The median survival times of the BORIS sf6^high^ group (n = 9) and BORIS sf6^low^ group (n = 43) were 26 weeks and 209 weeks, respectively. The log-rank test revealed a significantly worse prognosis for BORIS sf6^high^ cases (P = 0.0481). The hazard ratio of BORIS sf6^low^ cases was 0.4355 (95% confidence interval: 0.1062–0.9906).

To confirm the protein expression of BORIS sf6 in lung cancer tissues, BORIS sf6 specific polyclonal antibody was established. Specificity of the antibody was confirmed by Western blot (S1 Fig). A total of 52 lung cancer tissues were immunohistochemically stained and BORIS sf6 specific staining was observed in nucleus (Fig 3A and S2 Fig). BORIS sf6^low^ cases and BORIS sf6^high^ cases were divided at 30% positivity. BORIS sf6 expression rate was not related to patient’s age and histological subtypes; however, it was related to clinical stages and BORIS sf6high cases were significantly higher in advanced cases (Stage III and Stage IV) (Table 1). Furthermore, BORIS sf6^high^ cases were related to poorer prognosis than that in BORIS sf6^low^ cases (Fig 3B).

**Table 1:** Expression of BORIS sf6 and characteristics of lung cancer patients.

		BORIS sf6^high^	BORIS sf6^low^	P value
		≧ 30%	< 30%	
		*n* = 9	*n* = 43	
**Age (±SD)**		63.3± 7.7	65.9± 9.6	0.436
**Sex (%)**				0.810
	Male	5 (56)	22 (51)	
	Female	4 (44)	21 (49)	
**Histological subtype (%)**				0.911
	Adenocarcinoma	5 (56)	26 (61)	
	Squamous cell carcinoma	2 (22)	7 (16)	
	Others	2 (22)	10 (23)	
**Stage (%)**				0.035*
	Early stage (I, II)	5 (55)	37 (86)*	
	Advanced stage (III, IV)	4 (45)*	6 (14)	

Data: Mean ± standard deviation. Statistical analysis was performed by Chi-square test. Asterisks indicate statistical significant difference.

### Detection of BORIS sf6 peptide-specific CTL response in HLA-A2-positive donors

**Fig 4:**
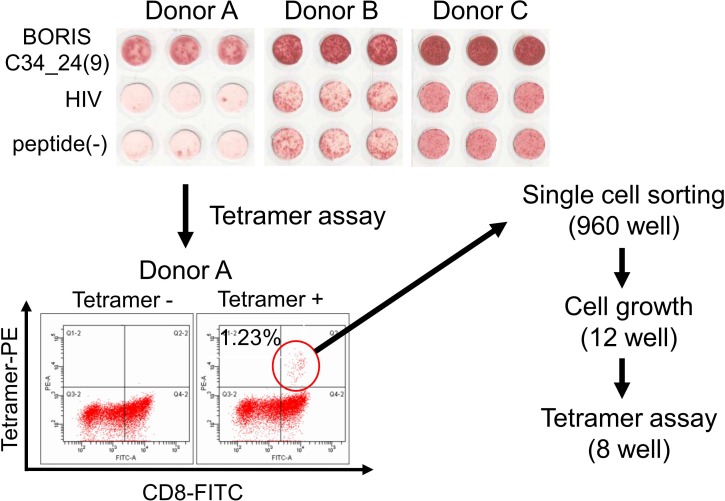
Induction of BORIS subfamily 6-specific CTLs and establishment of a CTL clone. BORIS subfamily 6 peptide-specific cytotoxic T cell (CTL) induction was evaluated by the interferon (IFN)-γ enzyme-linked immunospot (ELISPOT) assay. HLA-A*0201-positive PBMCs were obtained from four healthy donors. Donors A, B and C were HLA-*A0201-positive. Fluorescence-activated cell sorting (FACS) was performed with PE-conjugated BORIS subfamily 6 peptide/HLA-A*0201 tetramer and anti-CD8-FITC antibody. Double positive cells were single-cell sorted to establish a CTL clone (total 960 well). Twelve wells showed cell growth and 8 wells were analyzed by tetramer for specificity.

We previously identified the antigenic peptide BORIS C34_24(9), an HLA-A2 restricted antigenic peptide derived from a BORIS sf6-specific amino acid sequence [27]. In this study, we confirmed the antigenicity of BORIS C34_24(9) by several HLA-A2-positive healthy donors’ PBMCs. PBMCs of three HLA-A*0201-positive healthy donors were stimulated by BORIS C34_24(9) peptide, and the specific reactivity was evaluated by the IFN-γ ELISpot assay and tetramer assay. BORIS C34_24(9) peptide-specific IFN-γ secretion was detected in from PBMCs of all of the three donors (Fig 4). A PE-conjugated BORIS C34_24(9) peptide/HLA-A*0201 tetramer and FITC-labeled anti-CD8 antibody double-staining was performed. A BORIS C34_24(9) peptide-specific population was detected in PBMCs from donor A, and the ratio of tetramer-positive cells was 1.23% (Fig 3). To further analyze the BORIS C34_24(9) peptide-specific CTLs, single cell sorting of the tetramer-positive population was performed to establish BORIS C34_24(9) peptide-specific CTL clones.

### BORIS sf6 peptide-specific CTL clones recognize HLA-A2-positive and BORIS sf6-positive lung carcinoma cells

**Fig 5:**
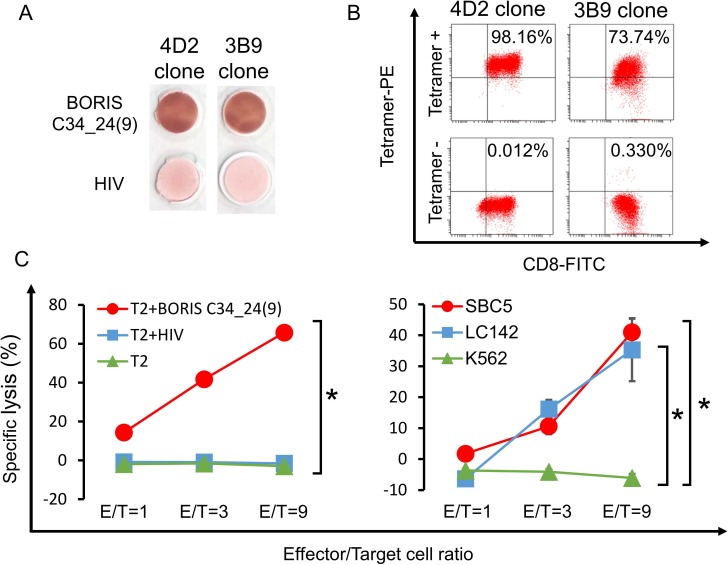
BORIS subfamily 6-specific CTL clone analysis. **(A) ELISPOT assay of BORIS subfamily 6-specific CTL clones 3B9 and 4D2 clones. (B) Tetramer assay of BORIS subfamily 6-specific CLT clones 3B9 and 4D2.** CTL clones 3B9 and 4D2 were stained by PE-conjugated BORIS subfamily 6-specific peptide/HLA-A*0201 tetramer and anti-CD8-FITC antibody and analyzed. **(C) LDH release cytotoxicity assay.** Specific cytotoxicity for peptide-pulsed T2 cells was displayed (left panel). HIV peptide-pulsed T2 cells, peptide (-) T2 cells and K562 cells were used as negative controls. Specific cytotoxicity for HLA-A2-positive lung cancer cell lines SBC5 and LC142 was displayed (right panel). Data are shown as means ± SD. All statistical analyses for data shown in this figure were performed using bilateral Student’s t test. *P-values <0.05.

Several CTL clones were established from single cell sorting culture, and an ELISpot assay was performed to confirm the specificity of CTL clones. Two CTL clones, 4D2 and 3B9, showed BORIS C34_24(9) peptide-specific IFN-γ (Fig 5A). The specificities of CTL clones were also confirmed by tetramer staining (Fig 5B). To confirm that these CTL clones could recognize lung carcinoma cells, we performed LDH release cytotoxicity assays. CTL clone 3B9 showed specific cytotoxicity for BORIS C34_24(9) peptide-pulsed T2 cells compared with that for control peptide-pulsed T2 cells or peptide (-) T2 cells (Fig 5C). CTL clone 3B9 showed significantly higher cytotoxicity for HLA-A2-positive and BORIS sf6-positive SBC5 and LC142 lung carcinoma cells than for HLA-A2-negative control K562 cells (Fig 5C). These results indicate that BORIS C34_24(9) peptide is endogenously expressed in HLA-A2-positive lung carcinoma cells.

**Fig 6:**
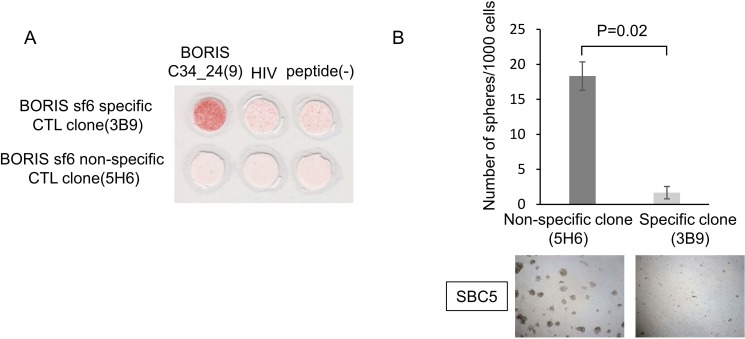
BORIS subfamily 6-specific CTL clone inhibited the sphere-formation of lung cancer cells. **(A) ELISPOT assay of BORIS subfamily 6-specific and non-specific CTL clones.** The reactivity for BORIS C34_24(9) peptide were analyzed by ELISPOT assay. HIV peptide and peptide (-) were used as negative controls. **(B) Sphere-formation inhibition assay.** BORIS C34_24(9) peptide specific CTL clone (3B9) or non-specific CTL clone (5H6) were co-cultured with SBC5 cells in a sphere-forming condition. After 6 days’ culture, the numbers of spheres were counted. Upper panel indicate the numbers of spheres, and data are shown as means ± SD. Lower panel indicates representative images of spheres. Magnification, x 100. Bar scale is 100 μm.

To further confirm whether BORIS C34_24(9) peptide-specific CTL clone can recognize subpopulation of lung CSCs/CICs, we performed sphere-formation inhibition assay. SBC5 cells or LC142 cells were co-cultured with BORIS C34_24(9) peptide-specific CTL clone 3B9 or BORIS C34_24(9) peptide non-specific CTL clone 5H6 (Fig 6A). CTL clone 3B9 significantly inhibited the sphere-formation of SBC5 cells compared with CTL clone 5H6 (Fig 6B). And similar result was obserbed in an experiment using LC142 cells.

## Discussion

Lung CSCs/CICs were isolated as SP cells, ALDH^high^ cells, CD133-positive cells or CD166-positive cells. In this study, we cultured lung cancer cells in a floating condition and could isolated spheres from all tested cells. The sphere-cultured cells showed expression of stem cell-related markers, indicating that lung CSCs/CICs are enriched in sphere-cultured cells. A previous study revealed that lung CSCs/CICs are enriched in sphere-cultured cells derived from lung cancer cell line H446 and A549 [28, 29]. Since the viability of primary lung carcinoma cells is not good, isolation of lung CSCs/CICs from primary lung carcinoma cells is difficult. In this study, we successfully isolated sphere-cultured cells from 4 lung cancer cases by sphere culture. Thus, sphere culture might be a good approach to isolate lung CSCs/CICs not only from lung cancer cell lines but also from primary lung carcinoma cells.

Progress in molecular analysis of lung cancer has enabled molecular targeted therapy of lung cancers, and some improvement has been achieved by using this therapy in lung cancer patients [30]. However, recent studies have revealed that lung CSCs/CICs are resistant to several molecular targeted therapies [14–16]. Therefore, establishment of a novel approach for lung CSCs/CICs is needed to improve the overall survival of lung cancer patients. In this study, we found that BORIS sf6 is expressed in lung adherent-cultured cells and also in lung sphere-cultured cells. BORIS has a role in the transcription of human telomerase in testicular and ovarian tumor cells [31], and BORIS is expressed in several CSCs/CICs and has a role in the maintenance of CSCs/CICs [27, 32, 33]. Thus, BORIS is a reasonable target for treatment-resistant CSCs/CICs.

Recent studies on lung cancer immunotherapy have revealed that treatment with an anti-programmed death-1 (PD-1) antibody improves the overall survival of patients with non-small cell lung cancers [17, 34, 35]. PD-1 is an inhibitory receptor that is expressed on cytotoxic T lymphocytes (CTLs) in the exhausted phase and suppress cytotoxic activity [36]. CTLs recognize antigenic peptides derived from tumor-associated antigens (TAAs) presented by major histocompatibility complex (MHC) class I molecules. Therefore, identification of antigenic peptides is essential for successful cancer-specific immunotherapy. We recently showed that treatment-resistant CSCs/CICs are susceptible to CTLs [37, 38]. Thus, CTL-based cancer immunotherapy might be a promising novel approach to target CSCs/CICs. BORIS is known as a cancer-testis antigen and it is immunogenic to humoral immunity and cellular immunity [27, 39]. A cancer-testis antigen is the first tumor-associated antigen found in human cancers [40]. Several cancer-testis antigens are preferentially expressed in CSCs/CICs [33, 41, 42], and cancer-testis antigens might thus be reasonable candidates for lung CSC/CIC-targeting immunotherapy. In this study, we observed that BORIS sf6-specific CTL clone could inhibit sphere-formation of lung cancer cells indicating that BORIS sf6-specific CTL clone recognized lung CSCs/CICs. Therefore, BORIS is a good candidate for lung CSC/CIC-targeted therapy. BORIS is ectopically expressed in other malignancies including glioblastoma [43], melanomas [44], head and neck squamous cell carcinoma [45], esophageal cancer [46], breast cancer [47], prostate cancer [48], uterine corpus cancer [49] and ovarian cancer [50], and BORIS might be a reasonable candidate for immunotherapies of those malignancies.

In summary, we found that the cancer testis antigen BORIS sf6 is preferentially expressed in lung CSCs/CICs. BORIS sf6 is expressed in lung cancer tissues. BORIS sf6-specific CTL clones could recognize HLA-A2-positive and BORIS sf6-positive lung carcinoma cells and inhibited the sphere-formation of lung cancer cells. The results indicate that BORIS sf6 is a reasonable candidate for treatment-resistant lung CSCs/CICs.

## Supporting information

S1 Fig Establishment of BORIS sf6 specific polyclonal antibody. 293T cells were transfected with Mock plasmid or myc-tagged BORIS sf6 DNA. Western blot using anti-myc or anti-BORIS sf6 antibody were performed. Anti-myc antibody was used at 0.2 μg/mL. Anti-BORIS sf6 antibody was used at 1000-times dilution.
(TIF)

S2 Fig Representative images of BORIS sf6 immunohistochemical staining. BORIS sf6^high^ cases are shown. Magnifications, 200 x and 630 x.
(TIF)
